# Systemic lupus pregnancies are characterized by an intrinsic pro-inflammatory monocyte transcriptome, driven by an aberrant miRNA signature

**DOI:** 10.1016/j.jtauto.2025.100347

**Published:** 2025-12-24

**Authors:** Marc Scherlinger, Eloi Schmauch, Raphaël Carapito, Angélique Pichot, Ghada Alsaleh, Nicodème Paul, Anne Molitor, François Lefebvre, Catherine Schmidt-Mutter, Seiamak Bahram, Jean Sibilia, Philippe Georgel

**Affiliations:** a Rheumatology Department, Centre National de Référence des Maladies Auto-Immunes et Systémiques Rares Est/Sud-Ouest (RESO, Hopitaux Universitaires de Strasbourg, 1 avenue Molière, 67098, Strasbourg, France; b Laboratoire d’ImmunoRhumatologie Moléculaire, Institut national de la santé et de la recherche médicale (INSERM) UMR_S 1109, Institut thématique interdisciplinaire (ITI) de Médecine de Précision de Strasbourg, Transplantex NG, Faculté de Médecine, Fédération Hospitalo-Universitaire OMICARE, Fédération de Médecine Translationnelle de Strasbourg (FMTS), Université de Strasbourg, Strasbourg, France; c Department of Public Health, University Hospital of Strasbourg, 67000, Strasbourg, France; d University Hospital of Strasbourg, CIC INSERM, 1434, Strasbourg, France; e UMR 7242, Biotechnologie et Signalisation Cellulaire, Team Neuroimmunologie et thérapie Peptidique ISIS, 8 All Gaspard Monge., 67000, Strasbourg, France

**Keywords:** Systemic lupus erythematosus, Rheumatoid arthritis, Pregnancy, Transcriptome, miRNA

## Abstract

**Objective:**

Pregnancy induces profound immunological adaptations that usually promote tolerance and reduce autoimmune activity. However, women with systemic lupus erythematosus (SLE) remain at increased risk of disease flares and pregnancy complications, whereas rheumatoid arthritis (RA) often improves during gestation. To better understand this divergence, we longitudinally characterized transcriptomic and microRNA (miRNA) changes in circulating monocytes from healthy, RA, and SLE pregnancies.

**Methods:**

Pregnant women with SLE (n = 5), RA (n = 4), and healthy controls (n = 5) were followed from preconception to three months postpartum. CD14^+^ monocytes were isolated at each visit and profiled using RNA sequencing and miRNA sequencing. Differential expression analyses were performed using DESeq2, modelling patient ID as a covariate. Pathway enrichment and upstream regulator analyses were conducted using Ingenuity Pathway Analysis and Reactome. Correlation-based miRNA–mRNA regulatory networks were inferred using miRTarBase-validated interactions.

**Results:**

Healthy and RA pregnancies exhibited a shift toward an alternatively activated (anti-inflammatory) monocyte phenotype, characterized by downregulation of TNF, IFN-γ, and IL-1 signalling pathways. In contrast, SLE pregnancies maintained a persistent M1-like (pro-inflammatory) program throughout gestation and postpartum, independent of clinical flare status. miRNA profiling revealed selective downregulation of miR-106a-5p and miR-148b-5p in SLE monocytes, accompanied by enrichment of cytokine-related pathways among their predicted targets. These dysregulated miRNAs were linked to activation of immune pathways including IL-12 signalling, interferon responses, apoptosis, and complement activation.

**Conclusion:**

SLE pregnancies are characterized by a failure to achieve monocyte immunotolerance, driven in part by aberrant miRNA regulation. These findings highlight molecular mechanisms underlying persistent inflammation in SLE pregnancy and identify candidate transcriptomic and miRNA biomarkers that may support future risk stratification or therapeutic modulation.

## Highlights

• SLE pregnancies are characterized by a persistent M1-like (inflammatory) monocyte transcriptomic signature, unlike healthy and RA pregnancies.
• Downregulation of specific miRNAs (miR-106a-5p, miR-148b-5p) may drive monocyte activation in SLE pregnancy.
• This suggests an intrinsic failure of immune adaptation in SLE pregnancy and identify potential molecular targets for monitoring or intervention.

## 1 Introduction

Systemic lupus erythematosus (SLE) is a potentially life-threatening autoimmune disease which mainly affects women of child-bearing age. SLE is a chronic disease during which low disease activity periods alternate with phases of higher activity (SLE flares) characterized by important inflammatory responses that may result in irreversible organ damage. Current recommendations aim at reaching clinical remission to prevent flares and organ damage [1,2]. SLE flares may be induced by UV exposure [3], infections [4,5] and even stress [6]. Among other identified triggers, pregnancies and post-partum state are known to be risk periods for SLE patients, and may be responsible for adverse pregnancy outcomes [7].

During pregnancy, the immune system enters a physiological immunosuppression state to prevent immune rejection of the foetus [8]. Theoretically, the pregnancy-associated immunosuppression state should improve autoimmunity and reduce clinical activity of autoimmune diseases. While this is the case for several autoimmune diseases such as rheumatoid arthritis or spondylarthritis [9], SLE pregnancies are associated with increased risk of flares [7]. However, the mechanism underlying this feature specific to SLE is largely unknown.

To monitor the state of the immune system during pregnancy, we conducted a longitudinal evaluation of healthy pregnant women as well as SLE and RA pregnant patients. We chose a whole genome transcriptomic approach (RNAseq) to globally evaluate changes in the expression of immune-related genes before and during pregnancy and after delivery. Transcriptomic analysis of lupus patients during pregnancy has been previously performed using RNA isolated from blood cells [10]. In the present study, we chose to focus on isolated circulating monocytes (CD14^+^) to better capture the transcriptomic features in a cell population known to be an essential player in the regulation of inflammatory responses [11]. To gain a better understanding of the mechanism driving transcriptomic (mRNAs) alterations during pregnancy, we also evaluated microRNA (miRNA, which have been described to play a role in SLE pathogenesis [12]) expression at each time point. Furthermore, dysregulation of several miRNAs (such as miR-142-3) in monocytes has been associated with increased inflammation in SLE patients [13], providing rationale to our unbiased exploration.

We found that SLE pregnancies are associated with an intrinsic M1-like monocyte transcriptome [14], partly driven by specific miRNAs. Our study suggests that a better description of the impact of pregnancy on the immune systems may help improving the follow-up of SLE pregnant women and promote the development of better tailored treatments for such patients.

## 2 Patients and methods

### 2.1 Patients and ethics

We included adult women under the age of 40 with a confirmed pregnancy (using β-HCG blood test). Three groups of pregnant women were included: healthy women without underlying autoimmune disease (HP), women with rheumatoid arthritis (RA) and women with SLE. Rheumatoid arthritis and SLE were diagnosed according to the ACR/EULAR revised classification criteria [15,16]. The clinical protocol is in line with the Helsinki declaration and was approved by an independent ethical committee (CPP Est IV, number DC 2013-1911). A written consent was obtained from all patients.

### 2.2 Follow-up and blood samples

Patients were included either prior pregnancy or at the time of a positive pregnancy test. Six to seven visits were conducted for all patients: an optional preconceptional visit (V0), a visit at the time of positive pregnancy test (V1), a visit at the end of the first trimester (V2), a visit at the end of the second trimester (V3), a visit during labour (V4), a visit one month (V5) and three months (V6) post-partum. At each time point, clinical data including the disease activity (SLE disease activity index [SLEDAI] for SLE and disease activity score 28 – CRP [DAS28-CRP] for RA) were recorded. For SLE patients, clinical activity was defined as an increase of 4 points in the SLEDAI. In RA, clinical activity was defined as a DAS28-CRP ≥3.2 (with the exclusion of infection).

### 2.3 Blood samples

At each visit, 3 EDTA-anticoagulated blood samples were collected and processed within 24 h. Peripheral blood mononuclear cells (PBMCs) were isolated using a Ficoll-Plaque gradient. Subsequently, monocytes were sorted using CD14-positive magnetic activated cell sorting (MACS, Miltenyi) according to the manufacturer's instruction. Total RNA was extracted from sorted monocytes using RNeasy microkit (Qiagen) according to the manufacturer's instructions.

### 2.4 Transcriptomics data collection and analysis

RNA-seq data were obtained using paired-end sequencing with a new generation sequencer SOLID 5500 (Life technologies). Sequencing data were processed using the nf-core/rnaseq pipeline, version 3.10.1 [17,18]. Within this pipeline, STAR was used for sequence alignment [19], while Salmon was used for RNA quantification [20], resulting in an expression matrix. For miRNAs, mirdeep2 was used to generate the expression matrix [21]. Downstream, DESeq2 was used to study differential expression for both RNA-seq and miRNA-seq data [22]. For each group (SLE, RA, or control), patient visits were ordered chronologically and modeled as a continuous variable to investigate transcriptional dynamics over visits. DESeq2 was run separately for each group with the design ∼ patient_id + visit_number, thereby treating patient ID as a covariate to account for inter-individual variability while exploring gene expression changes associated with progress of pregnancy visits. DESeq2's standard pipeline was used: size-factor normalization, dispersion estimation, and fitting a negative binomial GLM, followed by Wald tests on the visit number coefficient. P-values were adjusted using the Benjamini–Hochberg procedure to control the FDR (False discovery rate). For enrichment analyses and regulator predictions, Ingenuity Pathway Analysis (IPA) software was used to decipher the expression variations observed [23]. To link miRNAs and downstream mRNA targets, a correlation analysis was performed between mRNA and miRNA signals for each patient. A miRNA was predicted to negatively regulate a downstream mRNA if it was negatively correlated with that species across all samples (Pearson R < −0.5), and if predicted to target that mRNA by miRTarBase [24]. Gseapy was used to perform gene set enrichment analysis from the list of predicted targets of differentially expressed miRNAs [25].

## 3 Results

**Fig. 1:**
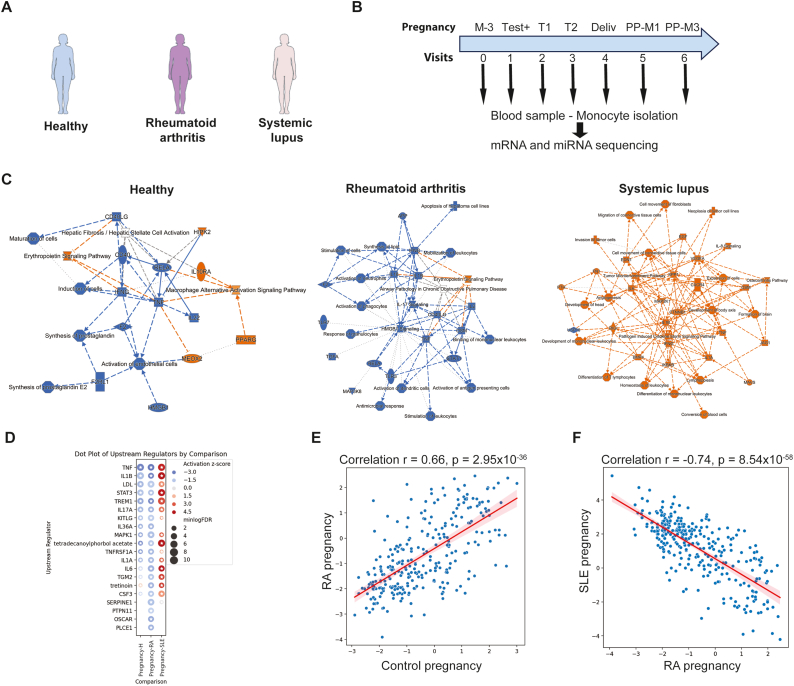
**SLE monocytesdemonstrate a pro-inflammatory signature during SLE pregnancy. (A)** Pregnant women recruited in the study: healthy (n = 5), rheumatoid arthritis (RA, n = 4), systemic lupus erythematosus (SLE, n = 5)**. (B)** Study design and timing of samples collection during pregnancy. **(C)** Ingenuity Pathway Analysis (IPA) showing upregulated (orange) or downregulated (blue) pathways throughout pregnancy in healthy, RA and SLE pregnancies**. (D)** IPA predicted upstream regulators activity during pregnancy. **(E**–**F)** Correlation between control and RA (E) and SLE and RA (F) pregnancies in terms of predicted upstream regulator activity**.**

Due to recruitment difficulties following the COVID-19 pandemic, 14 patients were recruited instead of 40 (SLE, n = 5; RA, n = 4; HD, n = 5; Fig. 1A). All patients completed at least 6 of the 7 visits (preconceptional visit 0 missing for n = 4; Fig. 1B). The average age was 32 (IQR 25–75: 28–32) for both HD RA and SLE women. Other patient characteristics including ongoing treatments are shown in Table 1. During pregnancy, two SLE patients had a non-severe flare: one during the first trimester and one three months postpartum. One RA patient exhibited clinical activity during pregnancy (between the positive test and the end of the first trimester, then three months postpartum) and one SLE patient flared immediately after giving birth (V6). All 14 pregnancies resulted in viable babies, without pre-eclampsia or other complications.

**Table 1:** Patients and pregnancies characteristics.

	Cohort (n = 14)	SLE (n = 5)	RA (n = 4)	Healthy (n = 5)
Age, median (IQR), years	30 (28–32)	32 (30–34)	32 (28–35)	28 (26–35)
BMI, median (IQR), kg/m^2^	21.6 (19.7–25)	27.3 (25.6–28.7)	19.6 (18.7–20.6)	20.2 (19.5–23.4)
Primiparity	71.4 %	40 %	75 %	100 %
Number of previous pregnancy, median (IQR)	0 (0–2)	1 (0–4)	0 (1–0)	0 (0–0)
Living birth	100 %	100 %	100 %	100 %
Disease flare during pregnancy	N/A	40 %	25 %	N/A
Treatments				
- Prednisone	64.3 %	100 %	100 %	0 %
- Aspirin	14.3 %	40 %	0 %	0 %
- Hydroxychloroquine	42.9 %	80 %	50 %	0 %
- Azathioprine	14.3 %	40 %	0 %	0 %
- TNF inhibitor	14.3 %	0 %	50 %	0 %

The mRNA transcriptome was determined for a total of 94 samples and the miRNome was evaluated in 69 samples. The discrepancy reflects the difficulty to isolate quality miRNAs. Quality assessment of the mRNA sequencing data indicated high sequencing quality across all samples, except for two that were excluded from downstream analysis (Fig. S1A).

Throughout pregnancy (V0-V7), regulatory networks analysis using Ingenuity Pathway Analysis (IPA) showed that monocytes (CD14^+^) from control and RA donors, although not superimposable, are characterized by an M2 polarization (anti-inflammatory) phenotype, as evidenced by a downregulation of the interferon gamma (IFNG), tumor necrosis factor (TNF), and HMGB1 pathways (Fig. 1C left and middle panel) over the course of visits (Tables S1–3). In comparison, monocytes from SLE donors (Fig. 1C, right panel) are characterized by an upregulation of the IFNG, TNF, interleukin 1 and interleukin 6 pathways, revealing a pro-inflammatory polarization. Importantly, the exclusion of the sample corresponding to the SLE patient with a flare did not affect this pro-inflammatory signature (Fig. S1B). Of note, CCL3L1 appeared significantly upregulated (Padj = 0.015, Table S3), which has been described as an important feature of M1-like monocytes [14,26].

Signaling pathway analyses demonstrated an attenuated pro-inflammatory signaling in RA and control pregnancies, with a significant correlation suggesting similar signaling changes (r = 0.82, p = 3.28x10^−6^; Fig. S1C–D). Conversely, there was a pro-inflammatory signaling in SLE pregnancies, and a trend of inverse correlation of these signaling pathway compared to RA patients (Fig. S1D, right panel). In agreement with these observations, upstream regulators analysis identified an upregulation of TNF, IL1B and IL-6 signaling in SLE pregnant patients while they were downregulated in control and RA (Fig. 1D). Interestingly, expression patterns of upstream regulators were highly correlated in control and RA pregnancies (Fig. 1E, r = 0.66, p = 2.95 x 10^−36^). In contrast, a negative correlation of upstream regulators between RA and SLE CD14^+^ cells was evidenced (Fig. 1F, r = - 0.74; p = 8.54 x10^−58^).

Altogether**,** these results suggest that during pregnancy, monocytes of SLE patients develop a pro-inflammatory transcriptomic phenotype which intrinsically differs from the situation seen in monocytes from healthy and RA pregnant women.

**Fig. 2:**
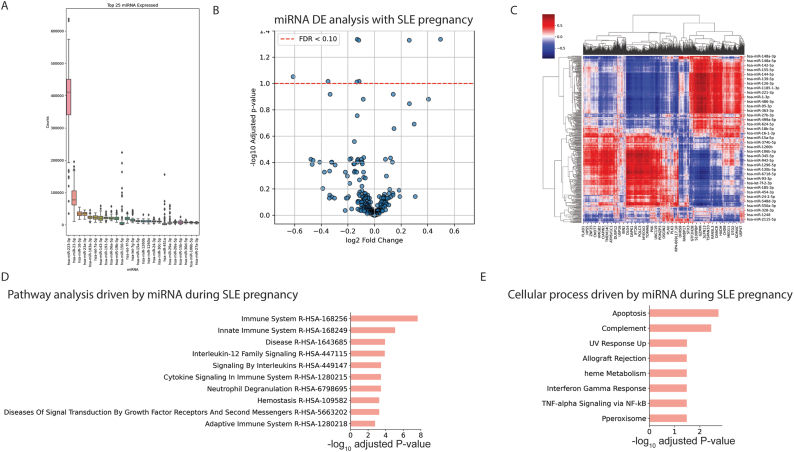
**In SLE pregnancy, miRNA accompany changes in monocyte transcriptomic signature**. (A) Top 25 most highly expressed miRNA across all samples. Counts are shown in counts per million (CPM). (B) Volcano plot showing the differential expression of miRNA across visits during SLE pregnancies. (C) Heatmap showing the correlation between mRNA expression and miRNA expression across samples. *(D) Reactome 2022* pathways predicted to be affected by miRNA in SLE pregnancies. *(E) GO Cellular process* gene-sets predicted to be affected by miRNA in SLE pregnancies. For D and E, gene set enrichment is performed on the list of genes that are predicted to be regulated by differentially expressed miRNAs in SLE pregnancies (FDR <0.01, LFC <0, across the visits).

To gain better insight into the origin of these differences, we quantified miRNA expression in all monocyte samples analysed. Indeed, miRNAs are major players involved in the control of gene expression [27], and several reports indicate that they participate in the regulation of monocyte functions in various inflammatory settings [28]. Among the miRNAs expressed in monocytes from pregnant healthy controls and from patients, miR-223-3p, miR-21-5p, miR-16-5p and miR-23a-3p exhibited the highest expression levels (Fig. 2A). During SLE pregnancy, we identified 2 overexpressed and 6 downregulated miRNAs over the course of visits (False discovery rate p-value <0.1, mean normalized DESEQ counts >20; Fig. 2B). A correlation analysis was used to predict the different targets genes of miRNAs (Fig. 2C).

We subsequently attempted to link these miRNA profiles with changes in mRNA expression. We did not find significant miRNA expression difference explaining modifications in mRNA expression levels between controls and RA samples (Tables S4–5). However, in SLE pregnancies, we observed a significant decrease of miR-106a-5p and 148b-5p expression over the course of pregnancy in monocytes (Table S6). We found a total of 6 miRNAs that were downregulated with pregnancy (with FDR <0.1, LFC – Log Fold Change <0 and mean normalized DESEQ counts >20) and performed gene set enrichment analysis on their predicted target genes, which interestingly revealed potential associations with overactivation of various pathways, such as cytokine signaling and the interleukin-12 pathway (Fig. 2D). Furthermore, altered miRNA expression in SLE monocytes also led to predicted activation of processes such as apoptosis, complement activation, interferon gamma response and ultraviolet response (Fig. 2E), which are common features observed in SLE patients. Of note, miR-106-5p has been shown to regulate the expression of *MAP3K14* and *PTEN* genes [29,30], both of which can exhibit potential relevance in the context of SLE due to their role in immune cell intracellular signaling [31].

Overall, these data suggest that change in monocyte miRNA expression may be driving the monocyte pro-inflammatory signature that we described in SLE pregnancies.

## 4 Discussion

In this prospective, longitudinal study, in which we followed a small number of patients, we suggest that pregnancies in women with systemic lupus erythematosus (SLE) are associated with an intrinsic M1-like, pro-inflammatory, monocyte transcriptomic signature, distinct from that observed in healthy or rheumatoid arthritis (RA) pregnancies. Furthermore, monocytes harvested from SLE patients exhibit a marked overexpression of *CCL3*, a feature of cells capable of chemotaxis and with immunoregulatory potentials [14,26]. Such observation, that will need to be confirmed by future single cell RNAseq analyses, suggest that this monocyte subpopulation which can potentially amplify an inflammatory microenvironment, might represent an important therapeutic target. Notably, this inflammatory polarization persists throughout gestation and postpartum and appears to be, at least in part, driven by dysregulated microRNA (miRNA) expression.

Our findings suggest an explanation for the paradoxical exacerbation of SLE during pregnancy, despite the well-known global immunosuppressive state that typically protects against autoimmune flares [8]. In contrast to RA, the symptoms of which often decrease during pregnancy due to elevated anti-inflammatory cytokines [9], SLE monocytes appear refractory to this immunotolerant milieu. This resistance is characterized by sustained activation of pro-inflammatory signaling pathways, including TNF, IFN-γ, IL-1, and IL-6.

An important observation is that this pro-inflammatory signature remained evident even when excluding patients with clinical flares, suggesting that it may represent a baseline immunological state in SLE pregnancies, rather than a secondary effect of disease activity. This aligns with recent transcriptomic studies showing persistent immune dysregulation in SLE patients even during clinically quiescent periods [10,11].

The accompanying miRNA profiling provides potential mechanistic insight. Specifically, the downregulation of miR-106a-5p and miR-148b-5p may contribute to derepression of inflammatory gene expression in SLE monocytes. Both miRNAs have been previously implicated in the regulation of immune responses: miR-106a targets IL-10 and STAT3, while miR-148b modulates TLR signaling and cytokine production [27,28]. Our results thus support a model in which dysregulated miRNAs could contribute to the failure of immune adaptation during SLE pregnancy.

Several limitations of this study must however be acknowledged. The small sample size, due in part to COVID-19-related recruitment challenges, limits statistical power and precludes subgroup analyses (e.g., by flare status, medication exposure). In addition, while we took care to isolate pure CD14^+^ monocytes, we cannot fully exclude contamination by CD14 ^low^ neutrophils, which have been implicated in SLE pathogenesis and share some inflammatory signatures [32]. Future studies could employ more refined phenotyping (e.g., single-cell RNAseq or surface marker co-staining) to rule this out. Likewise, the absence of functional validation (e.g., miRNA mimics/inhibitors, cytokine production assays) limits causal inference regarding the role of miRNAs as drivers of the observed transcriptomic changes.

Despite these limitations, our study provides the first direct evidence of a sustained pro-inflammatory monocyte program in SLE pregnancies and its potential miRNA-driven regulation. These findings may have clinical implications, suggesting that monocyte transcriptomic or miRNA profiling could serve as biomarkers to anticipate complications or guide therapy. In particular, the identification of miR-106a-5p and miR-148b-5p as potential upstream regulators of inflammation could inspire future therapeutic strategies to restore immune tolerance in SLE pregnancies.

In conclusion, this study reveals that SLE pregnancies are characterized by a sustained, cell-intrinsic M1-like, pro-inflammatory, program in circulating monocytes, contrasting with the anti-inflammatory phenotype observed in healthy and RA pregnancies. This transcriptional profile may be partly driven by a distinct miRNA signature, notably involving downregulation of miR-106a-5p and miR-148b-5p. These findings highlight the failure of immunological adaptation in SLE pregnancy and provide mechanistic insight into the heightened risk of disease flares and complications during this critical period.

## CRediT authorship contribution statement

**Marc Scherlinger:** Data curation, Validation, Visualization, Writing – original draft, Writing – review & editing. **Eloi Schmauch:** Data curation, Formal analysis, Writing – review & editing, Validation, Visualization. **Raphaël Carapito:** Investigation, Methodology, Writing – review & editing. **Angélique Pichot:** Investigation, Writing – review & editing. **Ghada Alsaleh:** Investigation, Methodology, Writing – review & editing. **Nicodème Paul:** Formal analysis, Methodology, Resources, Writing – review & editing. **Anne Molitor:** Project administration, Resources, Supervision, Writing – review & editing. **François Lefebvre:** Data curation, Formal analysis, Methodology, Writing – review & editing. **Catherine Schmidt-Mutter:** Investigation, Resources, Writing – review & editing. **Seiamak Bahram:** Project administration, Supervision, Writing – review & editing. **Jean Sibilia:** Conceptualization, Funding acquisition, Project administration, Resources, Supervision, Writing – review & editing. **Philippe Georgel:** Conceptualization, Formal analysis, Funding acquisition, Investigation, Methodology, Project administration, Supervision, Validation, Writing – original draft, Writing – review & editing.

## Disclosures

None to report.

## Fundings

This project (“SPIRALE”) was funded by a grant from the French Minister of Health (PRTS 2013–13-012) with Hopitaux Universitaires de Strasbourg as coordinator.

## Declaration of competing interest

The authors declare that they have no known competing financial interests or personal relationships that could have appeared to influence the work reported in this paper.

## Data Availability

Transcriptomics data will be released on a public repository followed publication.
