# Supplementary material for: Systemic lupus pregnancies are characterized by an intrinsic pro-inflammatory monocyte transcriptome, driven by an aberrant miRNA signature
Source: J Transl Autoimmun. 2025 Dec 24;12:100347. doi: 10.1016/j.jtauto.2025.100347 (PMC12810336; doi:10.1016/j.jtauto.2025.100347)
Supplement: Multimedia component 1 [file mmc1.docx]

**Supplementary data**

Systemic lupus pregnancies are characterized by an intrinsic pro-inflammatory monocyte signature driven by an aberrant miRNAs signature

**Figure S1**

1. FastQC quality check on mRNA samples. Two samples (TE0115_T_V4, AN0104_LES_V4) were excluded due to poor quality.
2. (left panel) Ingenuity pathway analysis showing upregulated (orange) or downregulated (blue) pathways throughout pregnancy of SLE pregnancies after excluding the sample corresponding to a patient with a flare (n = 1). (Right panel) Correlation between the initial analysis and the one excluding one SLE sample with flare.
3. IPA predicted upstream pathway analysis during pregnancy.
4. Correlation between control and RA (left panel) and RA and SLE (right panel) pregnancies in terms of predicted pathway analysis.

**
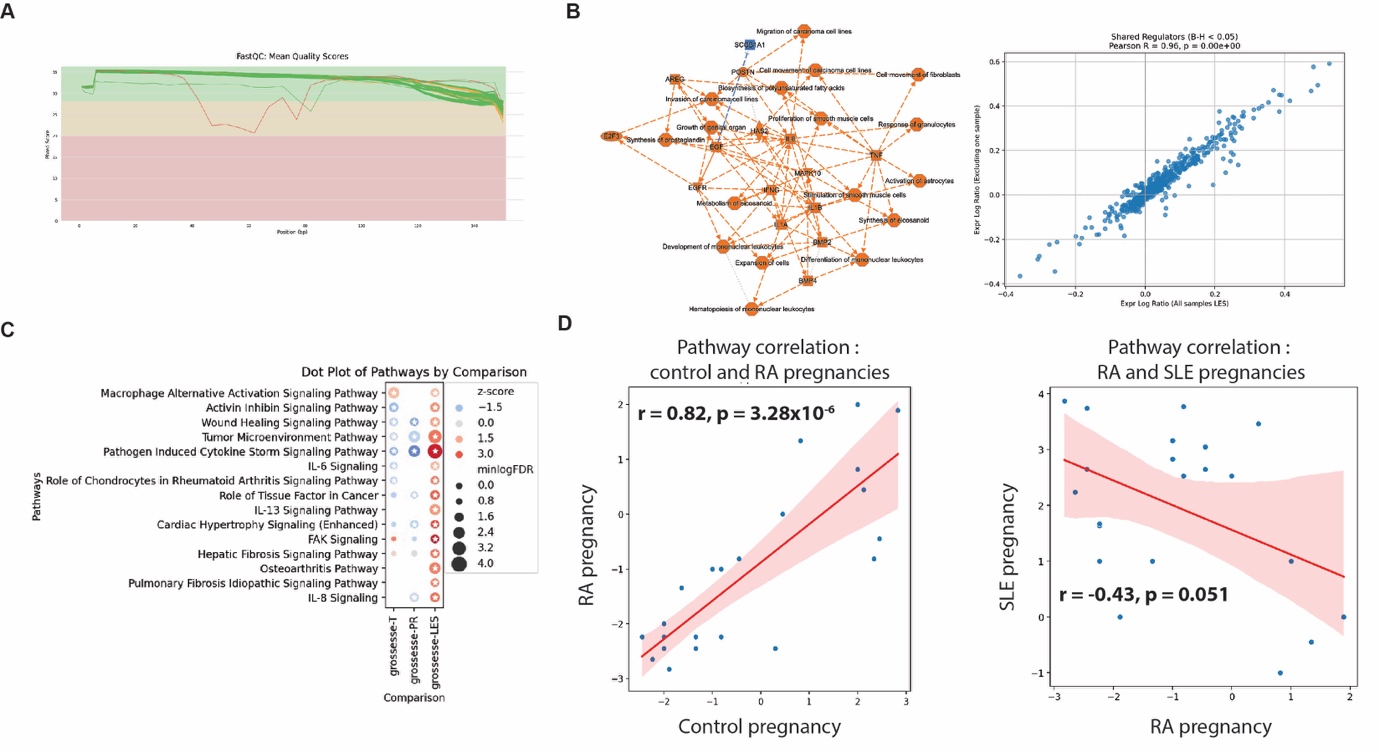
**

**Table S1: mRNA expression changes across visits in healthy pregnancies.**

DESeq2 Differential expression results.

**Table S2: mRNA expression changes across visits in rheumatoid arthritis pregnancies**

DESeq2 Differential expression results.

**Table S3: mRNA expression changes across visits in systemic lupus pregnancies**

DESeq2 Differential expression results.

**Table S4: miRNA expression changes across visits in healthy pregnancies.**

DESeq2 Differential expression results.

**Table S5: miRNA expression changes across visits in rheumatoid arthritis pregnancies**

DESeq2 Differential expression results.

**Table S6: miRNA expression changes across visits in systemic lupus pregnancies**

DESeq2 Differential expression results.
